# Evaluation of the Accuracy of Electronic Apex Locators in Modern Endodontics: An Umbrella Review

**DOI:** 10.3390/medicina60101709

**Published:** 2024-10-18

**Authors:** Massimo Pisano, Giuseppe Sangiovanni, Eugenio Frucci, Michela Scorziello, Giuseppina De Benedetto, Alfredo Iandolo

**Affiliations:** 1 Department of Medicine, Surgery and Dentistry, “Scuola Medica Salernitana”, University of Salerno, 84081 Salerno, Italy; eugenio.frucci@gmail.com (E.F.); scorziellomichela96@gmail.com (M.S.); giusydb15@gmail.com (G.D.B.); 2 Service of Maxillofacial Surgery, Stomatology and Hospital Odontology, CHU Besançon, 25030 Besançon, France; iandoloalfredo@libero.it; 3 Laboratoire Sinergies EA 4662, University of Franche-Comté, 25000 Besançon, France

**Keywords:** working length, root canal preparation, root canal therapy

## Abstract

*Background and Objectives:* To achieve success in endodontic treatment, it is essential to properly perform the steps of shaping, cleansing and obturation. Determining the working length of the canal is, therefore, a process that must be precise and accurate. Electronic apex locators are a useful tool for the clinician to best perform this step of endodontic treatment. *Materials and Methods:* The purpose of the following umbrella review is to evaluate, through data in the literature, the degree of accuracy of apex locators. *Results:* Seven systematic reviews were included in the following umbrella review. Five compare the accuracy of apex locators versus radiographic techniques, two compare different types of electronic apex locators, and two analyze the determination of working length in primary teeth. *Conclusions:* From the results obtained from the following umbrella review, albeit at low levels of evidence, the methods for determining working length using electronic apex locators and other methods, particularly using radiographic evaluation, are equally valid.

## 1. Introduction

The aim of endodontic treatment is to remove as much infected pulp tissue as possible, including the intracanal bacterial component, and affix an apical seal to prevent reinfection of the site [1]. To determine the success of an endodontic treatment, it is essential to comply with the following criteria: a proper access cavity, a proper shaping phase, and the three-dimensional cleansing and shaping of the complex root canal system. To perform proper contouring, cleansing, and obturation, it is necessary to accurately determine the working length [2,3]. Proper measurement of working length in endodontic treatment is a determining factor in long-term success and prognosis [4,5]. It is important to know the apical limit of the root canal. Kuttler in 1955 first studied the apical constriction or minor apical diameter. This is to be considered as the ideal limit for shaping and obturation of canals; in fact, it is the narrowest point with the smallest diameter and is generally 0.5–1.0 mm from the apical foramen [6]. The apical foramen or major apical diameter has variable morphology and size among different roots. During the root-forming phase, the apical foramen changes from being open, prior to the beginning of the maturation phase, to assuming a funnel shape due to the deposition of hard tissue. It may have an asymmetrical shape and thus consist of a larger and a smaller diameter. The apical foramen is not always located at the minor apical diameter. It has been observed that the distance between the major apical foramen and minor apical foramen is greater in posterior and older sector teeth than in anterior and younger sector teeth [7,8]. The anatomical apex represents the most apical point of the root term. It is usually straight, but with the passage of time, it tends to assume a distal curvature [9]. One needs to be able to distinguish the anatomic apex from the radiographic apex, which, on the other hand, is the most apical point of the root visible on radiography and does not always correspond to the anatomic apex. In addition, it is necessary to consider the degree of distortion of radiographic images, as well as the overlapping of other roots or anatomical structures, which can distort the measurement, compromising later stages of endodontic treatment [10]. Determining the correct working length is critical to achieve long-term clinical success and is a challenge that the dentist must know how to address. Protocols that involve determining working length by tactile perception or the use of radiographic aids are still used today. Such methods are unacceptable given the new technologies available. In endodontic treatments, the acquisition of radiographic information is through two-dimensional endoral radiographs that have distortions due to different factors such as the position of the tooth to be evaluated, the positioning of the radiographic film, and the position of the X-ray tube. The combination of these variables, which cannot be standardized, can lead to more or less significant image distortions that, therefore, do not allow for an accurate assessment of the working length [11]. To improve and make predictable the step of determining working length, electronic instruments have been developed over the years that exploit the physical concept of conduction to accurately determine root canal length. The first to present the idea that the working length of root canals could be evaluated by exploiting the physical characteristics of an electrical circuit was Cluster, in 1918 [12]. The first study conducted to support and evaluate the real effectiveness of Cluster’s idea was conducted by Suzuki [13] in 1942. Suzuki presented a machine that measured the electrical resistance formed between the periodontal ligament and the oral mucosa. He conducted animal studies that showed that, in dogs, there was a resistance produced between the metal file inserted into the canal and brought into contact with the periodontium and the electrode placed on the oral mucosa that was constant, with a value of about 6.5 kᾠ [13]. Electronic apex locators (EALs) take advantage of the characteristics of the patient’s body to enable the production of an electrical circuit. They consist of two ends that are positioned to induce the passage of electrical frequencies. The first component connects to an endodontic file inserted into the root canal via a clip mechanism. The second consists of a metal hook that is placed on the patient’s lip. The electrical circuit is produced when the file makes contact with the periodontal tissues present beyond the apex. Therefore, when the file goes beyond the apex, the electrical circuit is created, which is detected by the locator, which translates the electrical signal and converts it into an acoustic and visual signal on the display of the appliance, alerting the operator that it has gone beyond the apex. These devices have several advantages, chief among them being predictability and lowering of errors during the working-length determination phase. They are also very useful in those patients who present with a pronounced pharyngeal reflex and who poorly tolerate radiographic films or sensors [14].

In ex vivo studies conducted by ElAyouti et al., there are different variables that can alter and influence working length measurements using EAL such as apical constriction diameter, diameter and taper of the metal file inserted into the canal, presagulation, and the irrigation technique used in the cleansing phase [15]. Ni-Ti files that are inserted into preshaped canals can easily reach the apical third, presenting, thus, a lower incidence of overextension than files inserted into canals that have not been previously shaped [16]. For intraoperative evaluation of working length, .08 manual files or otherwise small-diameter files are almost always used. In certain clinical situations, such as wide canals or with immature or beating apices, the accuracy of EALs might be affected by the small size and small diameter of the file because a gap with the canal diameter is likely to be created. In these cases, a file with a larger diameter will have a tighter and therefore more accurate fit with the canal walls [17].

Although apex locators are found to have high accuracy, some factors can affect and lower the degree of accuracy in detecting working length. In particular, measurements are compromised by the presence of fluids such as blood, root canal irrigants, purulent tissue, and saliva within the canal as well as the presence of coronal metal restorations that do not allow the formation of an adequate electrical circuit [15]. Therefore, it becomes necessary to evaluate and compare the degree of accuracy of apex locators.

The following review aims to evaluate the degree of accuracy and precision of different apex locators based on the data reported in the literature, so as to enable clinicians to improve the predictability of endodontic treatments and provide dentists with the necessary elements to make an informed choice of working length detection technique and the most suitable apex locator.

## 2. Materials and Methods

### 2.1. Study Protocol

The PRISMA (Preferred Reporting Items for Systematic Reviews and Meta-analyses) protocol [18] was used to write the following umbrella review.

Studies were searched, selected and analyzed based on the Population–Exposure–Outcome (PEO) model [19], which is a modified version of the PICO model [20].

The PEO research focus was as follows:

P—Population: electronic apex locators.

E—Exposure: assessment of the degree of accuracy in the measurement of working length.

O—Outcome: comparison of working length with other measurement methods (radiographic and in vitro).

### 2.2. Search Strategy

The electronic literature search was carried out by two operators independently (G.S. and M.P.) and ended on 30 June 2024. Systematic reviews published in English were searched in the following search engines and databases: PROSPERO, MEDLINE/PubMed, Scopus and Cochrane Library.

The search was performed by combining the following keywords with Boolean operators:

(“Working length” or “Tooth Apex” or “Root Canal Preparation” [Mesh] OR “root canal length” OR “length determination”) and (“endodontic treatment” or “endodontics” or “root canal” or “root canal therapy” or “pulpotomy”).

“Review” and “Systematic Review” filters were applied and only articles published in English within the last 10 years, as of 2014, were included in order to obtain results that were not influenced by the analysis of obsolete EALs.

### 2.3. Eligibility Criteria

Before carrying out electronic document searches, inclusion and exclusion criteria were decreed in order to carry out a targeted search.

Inclusion criteria:

- Reviews with or without meta-analysis evaluating the degree of accuracy of EALs in determining working length in endodontic treatment through in vivo and in vitro studies;
- Systematic reviews with or without meta-analysis;
- Systematic reviews published since 2014;
- Systematic reviews published in the English language only.

Exclusion criteria:

- Reviews with or without meta-analysis that do not evaluate the degree of accuracy of EALs in determining working length in endodontic treatment;
- Clinical studies;
- Reviews published before 2014;
- Unpublished reviews in the English language.

### 2.4. Study Selection

In order to process and eliminate duplicates, the Zotero (version 7.0) reference manager tool was used, and the titles obtained from the search were analyzed by two authors independently (G.S. and M.P.); then, the abstracts of the titles obtained from the preliminary search were read and analyzed. The abstracts were then evaluated according to the degree of relevance to our review, and those of the studies found to be relevant were expanded by deriving the full text. The articles were screened by the two authors and a third reviewer (E.F.) was called in if there was any doubt or disagreement. Gray literature analysis was, in addition, conducted in order to identify reviews of interest for the following work. No restrictions were placed on the number and type of studies included in the systematic reviews analyzed.

### 2.5. Data Extraction and Collection

Data extraction was carried out by two authors (G.S. and M.P.) independently, and if there was no agreement between the two authors, a third (M.S.) was involved.

For each systematic review included in the following review, with or without the presence of a meta-analysis, the following data were recorded:

- First author of the review, year of publication, type of journal, and funding.
- Aim of study.
- Number and design of studies included in each systematic review and sample size.
- Type of EAL analyzed.
- Type of dentition in the sample (primary, mixed, secondary).
- Comparison method to assess measurement accuracy.
- Results.
- Conclusions.

### 2.6. Data Synthesis

To perform a qualitative synthesis of the included studies, a descriptive statistical analysis was performed using Microsoft Excel 2019 software, and then a narrative summary of the data on study population, exposure, and outcomes was produced.

### 2.7. Quality Assessment

The quality assessment of the systematic reviews presently included was performed through the Assessing the Methodological Quality of Systematic Reviews (AMSTAR) 2 tool, “https://amstar.ca (accessed on 19 August 2022)”, evaluating the systematic reviews of randomized and/or non-randomized studies for quality [20].

## 3. Results

### 3.1. Study Selection

A total of 3150 records were identified from the electronic search, specifically 854 from MEDLINE/PubMed, 261 from the Cochrane library, 720 from Prospero and 1315 from Scopus databases.

In total, 1658 duplicates were eliminated, 1479 title abstracts were screened.

Of these 2942 title abstracts, 1451 title abstracts were excluded and only 28 abstracts were relevant for this systematic review, so the full texts were screened and 21 articles were further excluded because 19 dealt with outdated EALs, while 2 studies were not systematic reviews.

**Figure 1:**
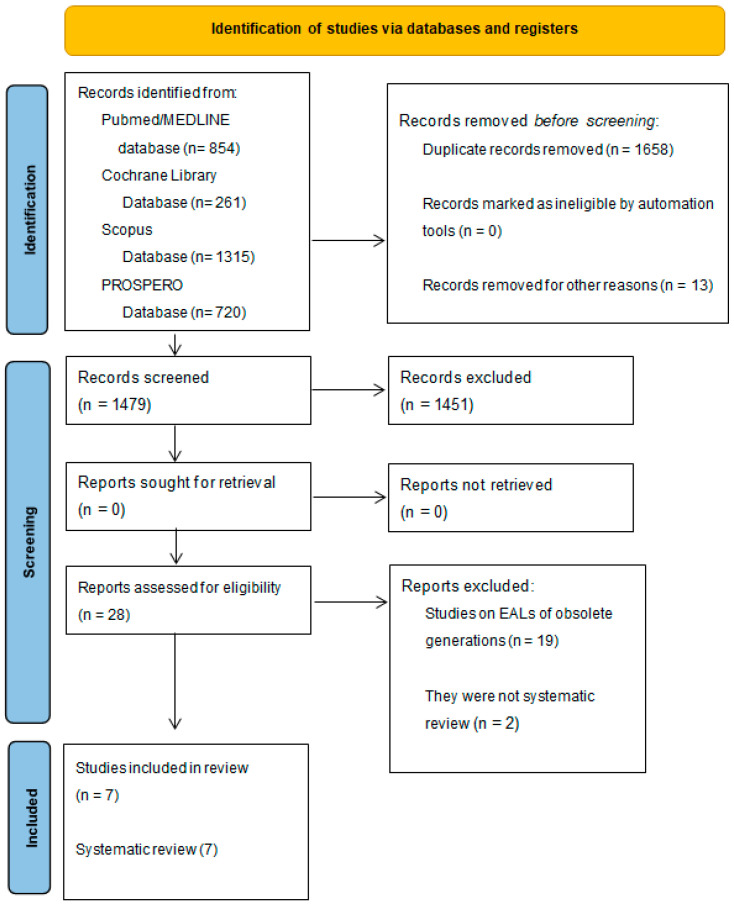
The study-selection flowchart.

The study-selection flowchart is illustrated in Figure 1.

A total of seven systematic reviews were finally included in the present umbrella review (Table 1).

### 3.2. Study Characteristics

The characteristics and outcomes from included studies are synthesized in Table 1.

**Table 1:** Data extracted from included studies: Source: first author, year of publication, bibliographic reference, journal or site of publication, meta-analysis, the presence or absence of funding. Studies reported in systematic reviews included in this general review: design and number; population sample size (no.), purpose; type of EAL analyzed; type of teeth in the sample, method of comparison to assess measurement accuracy, results, outcomes; conclusions.

-Authors -Year of Publication -Journal -Funding	-Design of Studies Included	-Aim	-Sample Size	Type of EAL Analyzed	Type of Teeth in the Sample (Primary or Permanent)	Comparison Method to Assess Measurement Accuracy	Outcomes	Results	Conclusions
Kaur [21] 2024 *BMC Oral Health* Non-funded.	clinical studies with cross-over and parallel-arm randomized controlled trials (RCTs)	The purpose of the systematic review with meta-analysis is to evaluate the effectiveness of EALs for determining working length compared with radiographic imaging.	Total Pz = 818 Total Root canals = 1262	- ROOT ZX—Third Generation - DENTA PORT ROOT ZX—Third generation - APEX ID—Third generation - IPEX II—Fourth Generation - Raypex5—Fourth Generation - RAYPEX-6—Sixth generation. - I-ROOT—Fifth generation. - Romiapex-A15—Third generation. - Sybron Endo mini apex locator—Third generation	Permanent	Periapical radiograph	Postoperative pain - Working length adequacy - Working length accuracy	- Pain: SMD 0.00 (CI -0.29, 0.28, 354 participants; *p* value = 0.98). - Working length adequacy: RR 1.10 (CI 1.03 to 1.18, 573 participants; *p* value = 0.02) - Working length accuracy: SMD 0.55 (CI 0.11 to 0.99, 254 participants; *p* value = 0.006)	There was no statistically significant difference in postoperative pain in the group undergoing apex locators compared with the radiographic group. The accuracy of working length is better in the group subjected to radiography while the adequacy of working length is better in the group where the electronic apex locator was used.
Paradiso [22] 2022 *Australian Endodontic Journal* /	- Clinical studies - in vivo study - in vitro study	The systematic review with meta-analysis aims to evaluate the degree of accuracy of determining working length in pulpectomy of primary teeth.	Total Pz = 356 Total canal roots =1701	- Root ZX - iPex - Propex Pixi - Foramatron D10 - EndoMaster	Primary teeth	- Tactile sensation (TS) - digital radiography (DR) - visual length (VL) (2.5x and 3.5x Magnification) - conventional radiography (CR)	Working length (mm; mean ± SD)	EAL and CR: OR was 0.73 (95% CI 0.48–1.11) EAL and DR Odds Ratio = 0.69 (95% CI 0.45–1.05)	The meta-analysis showed that the methods used to determine working length do not show statistically significant differences in the degree of accuracy.
Vitali [23] 2022 *International endodontic Journal* /	- randomized controlled trials (RCTs) - in vivo study - in vitro study	This systematic review with meta-analysis aims to evaluate the degree of accuracy of EALs in determining working length in pulpectomies in deciduous teeth.	Total Pz = 744 Total teeth = 1252 Total Canals = 2543	- Dentaport ZX - Propex II - Root ZX - Ipex - EndoMaster - Formatron D10 - R Smart Plus - NSK - Joypex 5	Primary teeth	- RVG - Visual direct - Conventional radiography - SEM	Working length	The overall pooled proportion for difference between electronic and comparative measures of <−1 mm was 1.97% (95% CI [0.66–3.96]), between −1 and −0.51 mm was 3.66% (95% CI [1.24–7.30]), between −0.5 and + 0.5 mm was 69.31% (95% CI [55.55–81.51]), between +0.51 and +1 mm was 18.20% (95% CI [11.23–26.41]), and >+1 mm was 5.31% (95% CI [1.07–12.52]).	The analysis in the following review shows acceptable accuracy of EALs in determining working length in pulpectomies of primary teeth but the included studies are of low quality. It is necessary, therefore, to evaluate these results with caution.
Martins [24] 2014 *Journal of Endodontics* /	- randomized controlled trials (RCTs) - in vivo study	The purpose of the systematic review was to evaluate the degree of accuracy in determining working length during endodontic treatment in adult patients with permanent dentition and to assess whether there was a reduction in radiation dose compared with the radiographic technique.	Total Pz = 2498 Total Teeth = 4819	- Tri-Auto ZX - Root ZX - DentaPort ZX - Raypex 5 - AFA Apex Finder Model 7005 - Bingo 1020 - Novapex - Neosono Ultima EZ - Apexpointer	Permanent	Conventional radiography	- Distance to the Radiographic Apex - Concordance of the Measurements by EAL When Compared with the Measurements of Radiography - Distance to the Apical Constriction, Cementodentinal Junction, and Apical Foramen.	- Distance to the Radiographic Apex range: 0.0 mm to 2.0 mm - Efficiencies for evaluating the distance to the Apical Constriction: 43.9–89.1% with EAL 14.6–32.72% with radiography	From the data from this review, it could be argued that the use of EAL is better than radiographic evaluation alone for determining working length in endodontic treatment.
Amin [25] 2019 *J Contemp Dent Pract* /	- in vivo study	The systematic literature review aimed to evaluate and compare the accuracy and reliability in determining working length between CBCT and EAL.	Total Pz = 82 Total Teeth = 166 Total Canals = 193	- J. Morita USA - J. Morita Japan - Dentsply, VDW	/	- CBCT	- Pearson correlation coefficient calculations of repeated measurements	- No difference, mean discrepancy between CBCT and EAL was 0.51 mm (CI = 95%)	While pointing out the limitations due to the heterogeneity of the included studies, it appeared on the limited available evidence that CBCT is as accurate as EAL in determining working length. There was a lack of high-level evidence comparing the reliability of CBCT with EAL. However, eligible studies have generally suggested that CBCT is reliable. New research will be needed in order to validate what has been observed.
Nasiri [26] 2022 *Saudi Dental Journal* /	- in vitro study - in vivo study	The aim of the work is to compare the degree of accuracy in measuring the working length of four different generations of EALs.	Total Canals = 966	- Apex ID - Elements Apex Locator - iPex - iPex II - Precision Apex Locator - Raypex 5 -Raypex 6 -ProPex Pixi - ProPex II - Root ZX (Dentaport ZX) - Root ZX II - Root ZX mini	Permanent	- the four generations of EALs	- Pearson correlation coefficient calculations of repeated measurements	heterogeneity tests: Q-values = 3.042 (3rd and 4th generation) Q-values = 4.569 (3rd and 5th) Q-values = 0.636 (4th and 5th) Q-values = 0.443 (3rd and 6th)	From the data obtained from the following systematic review, there are non-statistically significant differences among the four generations of apex locators analyzed in measuring working length.
Tsesis [27] 2015 *Journal of Endodontics*	- Ex vivo studies - in vivo studies	The purpose of this study was to evaluate the accuracy of EALs in localizing apical constriction and the effects of possible influencing factors through a systematic literature review and meta-analysis.	Total Canals = 1105	- Root ZX - Justy II - Endy 5000 - Endox	Permanent	- the four EALs	- Kruskal–Wallis tests with multiple comparisons (*p*-value < 0.05)	- The distance measured in the presence of NaOCl (*p* < 0.05) statistically smaller for Root ZX and Justy II. The distance between file tip and apical constriction did not differ statistically significantly between vital versus necrotic teeth.	The precision of electronic WL measurement depends on the device used and the type of irrigation and is not influenced by the status of the pulp tissue (vital or necrotic). Root ZX, Justy II, and Endy 5000 are significantly more precise than Endox. The precision of Root ZX and Justy II improves in the presence of H_2_O_2_ in comparison with the presence of NaOCl.

From the 3150 articles analyzed, 7 were eligible according to the inclusion criteria.

The most analyzed EALs were Root-ZX, iPex, iPex II, Raypex 5, and EndoMaster.

All articles consider in vivo studies; three also consider in vitro studies, and one includes ex vivo studies.

The total sample analyzed by the selected articles is divided into patients, teeth and canals. The total number of patients in the studies in the included reviews turns out to be 4498 results, while that of teeth analyzed is 6237. The total sample of canals, on the other hand, is 7770.

### 3.3. Elettronic Apex Locators vs. Radiographic Techniques

Five systematic reviews evaluated the degree of accuracy in determining working length during endodontic treatment by comparing the use of EALs and techniques using radiographs (Table 2).

### 3.4. Differences Between Different Types of Electronic Apex Locators

Two systematic reviews included within this umbrella review evaluated the degree of precision and accuracy in determining working length by different EALs with the aim of defining the most suitable for clinical needs (Table 3).

### 3.5. The Use of Electronic Apex Locators in Permanent and Primary Teeth

Another important parameter considered in this overview is accuracy in determining working length in permanent teeth and primary teeth (Table 4 and Table 5).

### 3.6. Quality Assessment

Most of the studies were judged to be of low or moderate quality through the Assessing the Methodological Quality of Systematic Reviews (AMSTAR) 2 tool, as shown in the table below (Table 6).

## 4. Discussion

The purpose of this overview is to evaluate, through analysis of data reported in the literature, the reliability and accuracy in determining working length during endodontic treatment. In particular, data on the use of electronic apex locators, which represent a useful tool for the practitioner in daily clinical practice, were evaluated through the analysis of the systematic reviews included in the paper. The numerous studies on the main electronic databases show the great interest of the scientific community in these instruments, which were created with the aim of speeding up and improving the stage of determining the working length in order to predictably and accurately perform the stages of shaping, cleansing and root canal obturation.

### 4.1. Accuracy of Electronic Apex Locators vs. Radiographic Techniques

Determining working length is a crucial step in achieving a favorable clinical outcome of endodontic treatment. It is obtained by measuring the distance between a coronal landmark, arbitrarily placed on a variable anatomical reference, and a point termed apical constriction (AC) located between the greater apical foramen and the lesser apical foramen [28,29]. Identification of the AC is very complex clinically because it is highly variable [29]. Furthermore, from the two-dimensional radiographic analysis, the overlapping of anatomical structures and the degree of image distortion make the determination of AC difficult to interpret [30]. In addition, several authors have raised the question about the amount and frequency of radiation exposure during the detection of working length during an endodontic treatment, as well as the time required for two-dimensional image acquisition during the session [31].

In this overview, of the selected studies, five evaluated and compared the degree of accuracy in determining working length of EALs with two- and three-dimensional radiographic techniques.

Mixed results have emerged from analyses of data in the various systematic reviews. In particular, Kaur et al. [21] compared the two methods in terms of postoperative pain, accuracy, and adequacy. Accuracy is defined by the degree of discordance of measurements with respect to a predetermined target, in this case the apical foramen [32]. To precisely define accuracy, however, a histologic evaluation is necessary because AC is difficult to assess by radiographic criteria.

Adequacy is defined as the assessment of the distance between the master cone of gutta-percha and the apical foramen in a reference range of 0 mm to 2 mm [21].

The meta-analysis found that the use of radiographic methods (periapical radiograph) make the determination of working length more accurate, while electronic apex locators are better in terms of adequacy. The authors, however, point out that these data do not represent reliable conclusions due to issues with bias, inconsistencies and imprecisions in the included studies because of the low quality they show.

Paradiso et al. [22] compared the use of electronic apex locators and radiographic techniques (digital radiography and conventional radiography) in determining working length in primary teeth. Quantitative analysis showed that the effectiveness of apex locators was similar to radiographic methods, with no evidence of statistically significant differences between the different protocols. The authors also evaluated the possibility of using cone-beam computed tomography (CBCT) to measure working length and found this method to be valid only when the patient undergoes three-dimensional radiographic examination for reasons unrelated to the performance of endodontic treatment [32,33,34].

Vitali et al. [23] conducted a meta-analysis of data in the literature in order to evaluate the reliability and accuracy of apical locators in primary teeth compared with the use of radiographic methods (RVG, conventional radiography, and SEM). Comparison of the average measured working lengths revealed statistically significant differences between electronic and radiographic measurements, with the latter being longer on average. However, this finding was statistically significant only for the canals of multi-rooted teeth, particularly molars, while in incisors there were no differences. Despite these differences, the electronic apex locators were found to be reliable in determining working length.

Martins et al. [24] compared radiographic methods (conventional radiography) and the use of only third-generation or later electronic apex locators, so as to exclude data obtained from obsolete and less accurate devices [35]. The included studies had limitations in methodology; in particular, most were comparative or evaluative studies that did not directly compare the two techniques, but rather performed radiographic confirmation of the electronic method of determining working length. Another limitation is due to the methodology for obtaining an apical reference by visible marks on the digital screen, including references to the acoustic signal or position of the file tip at 0.5 mm and 1 mm. Clinical factors that could potentially alter the efficacy of EALs such as root resorption, immature apex [36], or metal crown restorations were excluded. Although taking into account the limitations and bias due to the low methodological accuracy of the included studies, the data obtained showed that electronic apex locators are more accurate and reliable than the method with radiographic evaluation.

Amin et al. [25] conducted a systematic review in order to evaluate the degree of accuracy in determining working length by cone-beam computed tomography (CBCT) and EALs. Again, the included studies had a high degree of heterogeneity that made it impossible to determine the accuracy of CBCT versus EALs. Comparison of the data obtained between the two methods showed no statistically significant differences in accuracy, even taking into account the limitations of the evidence.

### 4.2. Differences Between Different Types of Electronic Apex Locators

In order to obtain as comprehensive and useful an overview as possible for the purpose of evaluating the degree of accuracy of EALs, two systematic reviews that evaluated the degree of accuracy of different types of locators by comparing them were included in the following umbrella review.

Specifically, Nasiri et al. [26] conducted a meta-analysis to evaluate the degree of accuracy in determining working length by comparing electronic apex locators of four different generations. Studies evaluating third-, fourth-, fifth- and sixth-generation apex locators were included, specifically, three studies on third-generation devices, four on fourth-generation devices, three on fifth-generation devices, and one on sixth-generation devices, also referred to as “modified fifth”. Comparison of the data from the included in vitro and in vivo studies showed no statistically significant differences between the four generations of apex locators analyzed on based the degree of accuracy for determining working length.

Tsesis et al. [27] compared the degree of accuracy of three electronic apex locators, namely Root ZX, Justy II, Endy 5000 and Endox. The meta-analysis conducted showed that Root ZX, Justy II and Endy 5000 are significantly more accurate in determining working length than the Endox device. Likewise, there were no statistically significant differences between Root ZX, Justy II and Endy 5000 in terms of accuracy, being equivalent and equally effective and predictable in clinical practice.

### 4.3. Differences Between Permanent and Primary Teeth

For the determination of working length, there are substantial differences in the method and especially in the possibility of operative biases in cases where such measurement is performed on dental elements of the primary series. It turns out to be of paramount importance this stage since overinstrumentation during the shaping stage, or extrusion beyond the apex of irrigants or filling material, could result in damage to the permanent dental germ; in addition, short instrumentation would lead to non-resolution of apical infection [37,38,39].

The physiological processes of root resorption and remodeling must also be taken into account, which make the determination of working length more difficult to achieve accurately [36]. Two reviews evaluating the ability and accuracy of working length determination in primary teeth were included in this umbrella review [22,23]. Neither, however, directly compared the differences between the use and accuracy of apex locators in primary and permanent teeth.

In particular, Paradiso et al. [22] evaluated and compared different methods of detecting working length in primary teeth, but did not evaluate them by comparing them with studies conducted on specimens formed from permanent teeth. The meta-analysis showed that the methods used to determine working length led to no statistically significant differences in the degree of accuracy in primary teeth.

Vitali et al. [23] evaluated and compared different methods of detecting working length in primary teeth, but did not evaluate them by comparing them with studies conducted on samples formed from permanent teeth. The meta-analysis conducted by analyzing the values of working lengths obtained by different methods showed different values between electronic and comparison measurements between −0.5 mm and +0.5 mm in 69.31 percent of cases and a difference between +0.5 mm and 1 mm in 18.20 percent. Differences in measurements concentrated between −0.5 and +0.5 mm were also the most common in the subgroup analysis for incisors (82.55%) and molars (62.94%). Electronic measurements with an accuracy of 0.5 mm or less from the anatomical apex are considered highly accurate according to several authors of papers in the literature [40,41].

### 4.4. Limitations of the Study and Distorted Quality of the Research

The reviews included in this overview have very different objectives, and not all reviews and evaluations are equal. The results and data discussed are for EALs compared with other methods, such as radiographic methods, but also comparing different types of EALs. However, problems arise with studies on EALs using the radiographic method as a comparison because the limits and landmark points for determining the accuracy of measuring working length are not standardized and vary from review to review and article to article included in the systematic review.

The result is that the information cannot be taken as certain and absolute, and the conclusions are of variable interpretation. From the quality assessment analysis, most of the studies were classified as of low or moderate quality, through the use of the Systematic Reviews Methodological Quality Assessment Tool (AMSTAR) 2 [30]. The partial quality of the included reviews and the lack of adequacy of the RCTs included in the reviewed reviews represent two other limitations of this systematic review. It should also be noted that the included reviews have different settings and have a moderate to high risk of bias.

## 5. Conclusions

EALs are a useful tool in order to achieve success in endodontic treatments.

Accuracy of Electronic Apex Locators vs. Radiographic techniques:

From the results obtained from the following umbrella review, albeit at low levels of evidence, the methods for determining working length using EALs and other methods, particularly that using radiographic evaluation, are equally valid.

Differences between permanent and primary teeth:

It cannot be determined whether there is a difference in the accuracy and precision of EALs used on permanent teeth and primary teeth.

Differences between different types of electronic apex locators:

More studies with higher levels of evidence are needed to determine with certainty which methods and detectors are best for determining working length.

## Figures and Tables

**Table 2:** Characteristics and accuracy of determination of working length outcomes from included studies. Source: first author, year; main results.

Authors	Radiographic Method	Main Results
Kaur, 2024 [21]	Periapical radiograph	The accuracy of working length is better in the group subjected to radiography while the adequacy of working length is better in the group where the electronic apex locator was used.
Paradiso, 2022 [22]	- Digital radiography - Conventional radiography	There were no statistically significant differences in determining working length between EALs and radiographic techniques.
Vitali, 2022 [23]	- RVG - Conventional radiography - SEM	There were no statistically significant differences in determining working length between EALs and radiographic techniques
Martins, 2014 [24]	Conventional radiography	The use of EALs is better than radiographic evaluation alone to determine working length in endodontic treatment.
Amin, 2019 [25]	CBCT	From the limited evidence available, it was found that CBCT is as accurate as EAL in determining working length.

**Table 3:** Characteristics and accuracy outcomes from included studies. Source: first author, year; main results.

Author	Main Results
Nasiri, 2022 [26]	There were no statistically significant differences among the four generations of apex locators analyzed in measuring working length.
Tsesis, 2015 [27]	Root ZX, Justy II, and Endy 5000 are significantly more precise than Endox.

**Table 4:** Included studies that looked at permanent teeth as the sample. Source: first author, year; main results.

Author	Discussion
Kaur, 2024 [21]	In permanent teeth, the radiographic technique is more accurate in determining working length while the technique using the EAL is more appropriate.
Martin, 2014 [24]	The use of EALs is better than radiographic evaluation alone to determine working length in endodontic treatment in permanent teeth.
Nasiri, 2022 [26]	No statistically significant differences were found among the four analyzed generations of apex locators in measuring the working length of root canal anatomical complexes of permanent dental elements.
Tsesis, 2015 [27]	Root ZX, Justy II and Endy 5000 are much more accurate than Endox for determining working length in endodontic treatments of permanent teeth.

**Table 5:** Included studies that looked at primary teeth as the sample. Source: first author, year; main results.

Author	Discussion
Paradiso, 2022 [22]	The meta-analysis showed that the methods used to determine working length led to no statistically significant differences in the degree of accuracy in primary teeth.
Vitali, 2022 [23]	Acceptable accuracy of EALs in determining working length in pulpectomies of primary teeth is observed, but the included studies are of low quality.

**Table 6:** Level of evidence of systematic reviews with meta-analysis included according to the AMSTAR 2 tool.

Studies Selected	Question and Inclusion	Protocol	Study Design	Comprehensive Search	Study Selection	Data Extraction	Excluded Study Justification	Included Study Details	Risk of Bias	Funding Sources	Statistical Methods	Risk of Bias in Meta-Analysis	Risk of Bias in Individual Studies	Explanation of Heterogeneity	Publication Bias	Conflict of Interest
Kaur 2024 [21]	Yes	Yes	Yes	Yes	Yes	No	Yes	No	Yes	N/A	Yes	Yes	No	Yes	Yes	Yes
Paradiso 2022 [22]	Yes	Yes	Yes	Yes	Yes	No	No	No	Yes	N/A	Yes	Yes	Yes	Yes	Yes	Yes
Vitali 2022 [23]	Yes	Yes	Yes	Yes	Yes	No	No	Yes	No	N/A	Yes	N/A	Yes	Yes	Yes	Yes
Martins 2014 [24]	Yes	Yes	Yes	Yes	Yes	No	No	No	Yes	N/A	Yes	Yes	Yes	Yes	Yes	Yes
Amin 2019 [25]	Yes	Yes	Yes	Yes	Yes	No	No	No	Yes	Yes	Yes	Yes	Yes	Yes	Yes	Yes
Nasiri 2020 [26]	Yes	Yes	Yes	Yes	Yes	No	No	No	Yes	Yes	Yes	Yes	Yes	Yes	Yes	Yes
Tsesi 2015 [27]	Yes	Yes	Yes	Yes	Yes	No	No	No	Yes	N/A	Yes	Yes	Yes	Yes	Yes	Yes

## References

[1] Kustarci A., Arslan D., Er K., Kocak S., Altunbas D. (2015). Comparison of various current electronic apex locators to determine the working length using the clearing technique. Niger. J. Clin. Pract..

[2] Adams N., Tomson P.L. (2014). Access cavity preparation. Br. Dent. J..

[3] Golvankar K., Kader M.A., Latheef A.A., Mohammed Ali A.B., Abullais S.S., Sibagathullah M. (2019). Comparison of accuracy in determining the root canal working length by using two generations of apex locators—An in vitro study. Open Access Maced. J. Med. Sci..

[4] de Morais A.L., de Alencar A.H., Estrela C.R., Decurcio D.A., Estrela C. (2016). Working length determination using cone-beam computed tomography, periapical radiography and electronic apex locator in teeth with apical periodontitis: A clinical study. Iran. Endod. J..

[5] Sadaf D., Ahmad M.Z. (2015). Accurate measurement of canal length during root canal treatment: An in vivo study. Int. J. Biomed. Sci..

[6] Diwanji A., Rathore A.S., Arora R., Dhar V., Madhusudan A., Doshi J. (2014). Working length determination of root canal of young permanent tooth: An in vitro study. Ann. Med. Health Sci. Res..

[7] Gordon M.P.J., Chandler N.P. (2004). Electronic apex locators. Int. Endod. J..

[8] Martos J., Ferrer-Luque C.M., González-Rodríguez M.P., Castro L.A. (2009). Topographical evaluation of the major apical foramen in permanent human teeth. Int. Endod. J..

[9] Kuttler Y. (1955). Microscopic investigation of root apexes. J. Am. Dent. Assoc..

[10] Orosco F.A., Bernardineli N., Garcia R.B., Bramante C.M., Duarte M.A.H., Moraes I.G.d. (2012). In vivo accuracy of conventional and digital radiographic methods in confirming root canal working length determination by Root ZX. J. Appl. Oral Sci..

[11] Patel B. (2015). Endodontic radiology. Endodontic Diagnosis, Pathology, and Treatment Planning.

[12] Cluster L.E. (1918). Exact methods of locating the apical foramen. J. Natl. Dent. Assoc..

[13] Suzuki K. (1942). Experimental study on iontophoresis. J. Jpn. Stomatol..

[14] Pisano M., Bramanti A., Menditti D., Sangiovanni G., Santoro R., Amato A. (2023). Modern Approaches to Providing Telematics Oral Health Services in Pediatric Dentistry: A Narrative Review. Appl. Sci..

[15] ElAyouti A., Kimionis I., Chu A.L., Löst C. (2005). Exact determination of the working length by electronic apex locators, digital radiology, and visual test: Presentation of a new experimental research strategy—An ex-vivo study. Int. Endod. J..

[16] Pecora J.D., Capelli A., Guerisoli D.M., Spano J.C., Estrela C. (2005). Influence of cervical pre-flaring on apical file size determination. Int. Endod. J..

[17] Saritha V., Raghu H., Kumar T.H., Totad S., Kamatagi L., Saraf P.A. (2021). The accuracy of two electronic apex locators on effect of preflaring and file size: An in vitro study. J. Conserv. Dent..

[18] Khan L.K., Kunz R., Kleijnen J., Antes G. (2004). Systematic reviews to support evidence-based medicine. How to review and apply findings of healthcare research. Kahn, K.S.; Kunz, R.; Kleijnen J, Antes, G.170 × 240 mm. Pp.136. Illustrated.2003. Royal Society of Medicine Press: London, UK. Br. J. Surg..

[19] Richardson W.S., Wilson M.C., Nishikawa J., Hayward R.S. (1995). The well-built clinical question: A key to evidence-based decisions. ACP J. Club.

[20] Shea B.J., Reeves B.C., Wells G., Thuku M., Hamel C., Moran J., Moher D., Tugwell P., Welch V., Kristjansson E. (2017). AMSTAR 2: A critical appraisal tool for systematic reviews that include randomised or non-randomised studies of healthcare interventions, or both. BMJ.

[21] Kaur G., Thomas A.R., Samson R.S., Varghese E., Ponraj R.R., Nagraj S.K., Shrivastava D., Algarni H.A., Siddiqui A.Y., Alothmani O.S. (2024). Efficacy of electronic apex locators in comparison with intraoral radiographs in working length determination- a systematic review and meta-analysis. BMC Oral Health.

[22] Paradiso D., Tullio A., Bensi C. (2023). Working length determination in primary teeth pulpectomy: A systematic review and meta-analysis. Aust. Endod. J..

[23] Vitali F.C., Santos P.S., Cardoso M., Massignan C., Garcia L.D.F.R., Bortoluzzi E.A., Teixeira C.D.S. (2022). Are electronic apex locators accurate in determining working length in primary teeth pulpectomies? A systematic review and meta-analysis of clinical studies. Int. Endod. J..

[24] Martins J.N., Marques D., Mata A., Caramês J. (2014). Clinical efficacy of electronic apex locators: Systematic review. J. Endod..

[25] Amin J., Lines J., Milosevic M.P., Park A., Sholapurkar A. (2019). Comparison of Accuracy and Reliability of Working Length Determination Using Cone Beam Computed Tomography and Electronic Apex Locator: A Systematic Review. J. Contemp. Dent. Pract..

[26] Nasiri K., Wrbas K.T. (2022). Accuracy of different generations of apex locators in determining working length; a systematic review and meta-analysis. Saudi Dent. J..

[27] Tsesis I., Blazer T., Ben-Izhack G., Taschieri S., Del Fabbro M., Corbella S., Rosen E. (2015). The Precision of Electronic Apex Locators in Working Length Determination: A Systematic Review and Meta-analysis of the Literature. J. Endod..

[28] Iandolo A., Pisano M., Scelza G., Abdellatif D., Martina S. (2022). Three-Dimensional Evaluation of the Root Apex of Permanent Maxillary Premolars: A Multicentric Study. Appl. Sci..

[29] Abdellatif D., Iandolo A., Scorziello M., Sangiovanni G., Pisano M. (2024). Cyclic Fatigue of Different Ni-Ti Endodontic Rotary File Alloys: A Comprehensive Review. Bioengineering.

[30] Srivastava K.C., Shrivastava D., Austin R.D. (2016). Journey towards the 3D dental imaging-the milestones in the advancement of dental imaging. Int. J. Adv. Res..

[31] Grove C.J. (1922). Further evidence that root canals can be filled to the dentinocemental junction. J. Am. Dent. Assoc..

[32] Kayabasi M., Oznurhan F. (2020). Evaluation of the accuracy of electronic apex locators, cone-beam computed tomography, and radiovisiography in primary teeth: An in vitro study. Microsc. Res. Tech..

[33] Pantaleo G., Amato A., Iandolo A., Abdellatif D., Di Spirito F., Caggiano M., Pisano M., Blasi A., Fornara R., Amato M. (2022). Two-Year Healing Success Rates after Endodontic Treatment Using 3D Cleaning Technique: A Prospective Multicenter Clinical Study. J. Clin. Med..

[34] Iandolo A., Pisano M., Abdellatif D., Sangiovanni G., Pantaleo G., Martina S., Amato A. (2023). Smear Layer and Debris Removal from Root Canals Comparing Traditional Syringe Irrigation and 3D Cleaning: An Ex Vivo Study. J. Clin. Med..

[35] Abdellatif D., Iandolo A., De Benedetto G., Giordano F., Mancino D., Euvrard E., Pisano M. (2024). Pulp regeneration treatment using different bioactive materials in permanent teeth of pediatric subjects. J. Conserv. Dent. Endod..

[36] Leonardo M.R., Silva L.A., Nelson-Filho P., Silva R.A., Raffaini M.S. (2008). Ex vivo evaluation of the accuracy of two electronic apex locators during root canal length determination in primary teeth. Int. Endod. J..

[37] Iandolo A., Pisano M., Abdellatif D., Amato A., Giordano F., Buonavoglia A., Sangiovanni G., Caggiano M. (2023). Effectiveness of Different Irrigation Techniques on Post Space Smear Layer Removal: SEM Evaluation. Prosthesis.

[38] Oznurhan F., Ünal M., Kapdan A., Ozturk C., Aksoy S. (2015). Clinical evaluation of apex locator and radiography in primary teeth. Int. J. Paediatr. Dent..

[39] Capuano N., Amato A., Dell’Annunziata F., Giordano F., Folliero V., Di Spirito F., More P.R., De Filippis A., Martina S., Amato M. (2023). Nanoparticles and Their Antibacterial Application in Endodontics. Antibiotics.

[40] Balaji K., Pravallika T.S. (2019). A comparative evaluation of the accuracy of two electronic apex locators and radiovisiography to determine the working length. World J. Dent..

[41] Caliskan S., Delikan E., Cantekin K. (2021). Evaluation of methods for determining working length in root canal treatment for primary molars: An in-vivo study. Cyprus J. Med. Sci..

